# The impact of specialised translator training and professional experience on legal translation quality assurance: an empirical study of revision performance

**DOI:** 10.1080/1750399X.2024.2344948

**Published:** 2024-04-28

**Authors:** Fernando Prieto Ramos, Diego Guzmán

**Affiliations:** Centre for Legal and Institutional Translation Studies (Transius), Faculty of Translation and Interpreting, University of Geneva, Geneva, Switzerland

**Keywords:** Legal translator training, legal translation, legal translation competence, revision, translation quality assurance, ISO 20771:2020

## Abstract

The relevance of translation and law degrees as pathways to professional legal translation is the subject of persistent debate, but there is limited research on the relationship between legal translators’ backgrounds and competence levels in practice. This study compares the revision performance of several groups of institutional translators (44 in total) according to their academic backgrounds (legal translation specialisation, translation degrees with no legal specialisation, law degrees or other degrees) and legal translation experience (more or less than three years). The scores of justified, missing and over-corrections, among other indicators, corroborate the crucial impact of legal translation specialisation and subject-matter knowledge in ensuring legal translation quality, while experience can serve to partially fill certain training deficits. Qualified translators with a legal specialisation stood out as the most efficient revisers, followed by law graduates, translation graduates without a legal specialisation and other translators. A subsequent holistic assessment of the revised target text yielded results globally in line with the revision scores, as well as mixed perceptions of the target text as potential machine output. The findings support the added value of legal translator training, and are of relevance for translator recruitment and workflow management. They also challenge the rationale behind ISO 20771:2020 qualification requirements.

## 1. Introduction

As in other areas of specialised translation, pathways to professional practice in legal translation can be diverse. They ideally involve a combination of training oriented to the development of legal translation competence (LTC), including knowledge of law and legal language and translation methodology and practice. There is consensus about the distinctive nature and relevance of legal knowledge as core element of thematic competence in legal translator training, but the level of legal expertise required is the subject of a persistent debate in the field. Most scholars agree that an academic qualification in law and some knowledge of the languages involved are insufficient to ensure quality in professional legal translation, but at least some training in translation is necessary (e.g. Gémar 1988, 306; Scott 2017, 232; Sparer 2002, 275). Many authors argue that the legal translator should be primarily trained as a translator with specialisation in legal translation (e.g. Cao 2007, 39; Lavoie 2003, 394–395). Ideally, such training should be interdisciplinary and comprehensive, and encompass sufficient law courses for the efficient development and application of subject-matter knowledge in legal translation practice (Prieto Ramos 2011, 18–19; Šarčević 1997, 113–115; Soriano Barabino 2016, 152–153).

Given the diversity of individual pathways, programmes and profiles, it is difficult to determine the best combination and sequence of training types and levels, especially when appropriate legal translation programmes are not available at postgraduate level for a specific language pair. The assumption that law graduates are better candidates for legal translation than translation graduates was widespread before the proliferation of translation programmes. At the Court of Justice of the European Union (CJEU), for example, a law degree is required to work as a legal translator (with the job title of ‘lawyer-linguist’) (on recent recruitment practices, see Prieto Ramos and Guzmán 2022), while the recent ISO standard for legal translation, ISO 20771:2020 (ISO 2020), implicitly suggests that a law degree qualifies more directly than a translation degree for professional legal translation. It establishes an additional requirement of ‘the equivalent of at least three years’ full-time professional experience in translating documents within the legal field’ for compliance in the case of law graduates (ISO 2020, 9), while the same level of experience plus a postgraduate degree in law is required for translation graduates. Without such postgraduate training, and regardless of the specialisation pursued as part of a translation degree, the standard assigns the same value to translation degrees as to degrees in ‘any field’ by asking for five years of professional experience in legal translation (ISO 2020, 9).

For those qualified ‘as a certified legal translator from an officially recognised professional organisation’, regardless of the initial degree specialisation, the experience requirement is down to three years, i.e. a professional certification is more highly valued than a university degree with a focus on legal translation, which surprisingly is not acknowledged as a variation of training scenarios within the ISO standard. No experience is even required when the translator ‘has obtained an officially recognised qualification as an authorised legal translator on the basis of relevant national requirements and regulations’.

The project leader for ISO 20771:2020 asserts that ISO translation standards ‘address the issue of competence validation and minimum professional competence-based qualifications of a translator and define the different options in a consistent, clear and objective way’ (Popiołek 2020, 35). Yet, the qualification requirements set in ISO 20771:2020 are not supported by any empirical validation or objective data about performance levels.

In fact, there is little empirical research on the connections between legal translators’ backgrounds (including academic qualifications and professional experience) and their competence levels in practice. Among the most relevant studies, Orlando (2017) asked 15 translation graduates (from the same university) with no legal specialisation and 15 law graduates (from another university) with a demonstrated knowledge of English, but no translation background, to translate a European arrest warrant from English into Italian, in order to compare their performance. The better results obtained by the first group suggest that, prior to any academic specialisation or experience in legal translation, a translation graduate is equipped with more relevant competences for professional translation in the field than a law graduate with a good command of the source language and (presumably) the target language.

In another study based on the methodology applied by Künzli (2007), Pontrandolfo (2017) compared the Spanish-Italian revisions carried out by (1) five lawyer-linguists from the CJEU with 5 to 10 years of translation and revision experience, (2) five translators trained in legal translation but with less experience (1–5 years), and (3) five professional translators with 5–12 years of experience but no legal translation training. The text chosen was a judgement from a Spanish criminal court, which partially relates to the translation of judgements at the CJEU. Given the combination of translation specialisation and experience, it was not surprising that the first group excelled in the exercise, while the third group did better than those trained in legal translation, which points to the relevance of experience for competence development in professional settings. In this study, however, the disparity of experience levels within and between profile groups makes it very difficult to draw conclusions about the differential impacts of training and experience on performance.

Our study aims at filling a gap in the field by analysing the revision performance of several groups of professional translators classified according to their academic background and level of experience, with a view to measuring the potential impact of these variables on legal translation quality assurance (LTQA). The ultimate aim is to obtain empirical data on the benefits of specialised training and how these compare to competence development through practical experience. The study also examines variations in detecting legal content issues *versus* other issues, depending on the level of legal, translation or linguistic expertise among the diverse profile groups. The design and implementation of the study will be explained in more detail in the next section (Section 2). This will be followed by a summary of the results (Section 3) and the concluding discussion (Section 4).

## 2. Study design and implementation

Our study was designed as part of the last phase of the LETRINT project,^1^ which examines legal translation practices and LTQA in international institutional settings, including aspects related to competence, process and product (Prieto Ramos 2015). More particularly, the focus of this research is on the relationship between LTC and translation quality evaluation through the correlational analysis of several indicators: academic background and years of experience in legal translation (as variables associated to profile specialisation), and revision performance patterns, including multiple correction scores.

Revision was chosen not only because it is a key activity required of today’s translators in institutional settings, but also because of its commonalities with post-editing in calling for sound translation competence to detect and fix issues and improve the target text. As noted by Konttinen et al. (2020, 202), revision and post-editing are increasingly embedded in translation workflows through software tools, and their boundaries are not always clear when the origin of the text or segment is not known by the translator or reviser. The LETRINT project was interested in exploring this aspect as part of analyses of the perceived impact of technological advances on current working methods, and so a hybrid revision/post-editing exercise was considered. However, this would have entailed a number of technical constraints and difficulties to preserve ‘institutionally-neutral’ conditions for our measurements, and would involve availing of specific tailored tools, MT systems or translation memories.

A revision exercise was finally designed to explore (1) the extent to which participants had the ability to spot and correct errors, and whether their corrections were either justified or unnecessary; and (2) the extent to which profile variations associated to legal domain and legal translation specialisation play a role in correcting errors of a legal nature or requiring adequate knowledge of legal subjects or legal discourses. As subsidiary objectives, the study sought to gather information on the times devoted to the revision exercise and on the overall perception of the quality of the revised translation and how it had been initially generated. The revision brief would actually not reveal the human or machine origin of the text to be revised.

### 2.1. Text selection and error analysis

In light of the previous phases of the project and the aims of the study, a number of requirements were established to select an appropriate text for the revision tests:

1. The source text (ST) would have to be sufficiently representative of legal translation in international institutional settings in terms of genre, content and translation difficulties, while the target text (TT) would illustrate typical errors in the same context. The exploratory process to meet this requirement was supported through various preliminary LETRINT studies, including a mapping of legal genres translated in institutional settings (Prieto Ramos and Guzmán 2021), the scrutiny of features (especially, terminology and phraseology, and their associated translation difficulty levels) and translation errors spotted during the annotation of the parallel LETRINT 1+ corpus (see Prieto Ramos and Cerutti 2021 for more details on the corpus and the annotation process) and the analysis of errors typically rectified through institutional corrigenda (Prieto Ramos and Morales Moreno 2019).
2. Despite the representativeness of the text, the ST would not be associated with a standardised or prototypical genre of any particular institution, so that the revision test would not constitute an easier task for a particular group of participants due to affiliation. The aim was to examine the impact of specialised training and professional experience, avoiding institutional bias to the extent possible. In other words, the revision exercise would be geared towards testing individual competence in the field regardless of advanced acquaintance with particular institutional genres or sources. The ability to correct errors would be more associated with proficiency in legal translation and familiarity with essential legal notions.
3. The TT would contain a significant density of errors to be considered of poor quality for an institutional publication and to be associated with a low level of LTC. This would offer a wider range of possibilities for testing error detection among participants, and also for exploring their impressions of the human or machine origin of the TT, as the overall translation quality and error types would not necessarily make it easy to guess such origin.
4. The text length for revision would be a reasonable one for a total revision time of approximately 75 minutes, so as to secure adequate attention from participants. As demonstrated by research, factors such as duration, time constraints and other extratextual conditions can influence revision performance (see e.g. Künzli 2007).
5. English-Spanish translations would be considered for practical and quality assurance reasons. This language combination is found in the institutional settings considered, including IGOs and EU institutions, and was the one chosen in the LETRINT project for a number of language-specific studies, together with English-French and, to a lesser extent, French-Spanish translations. English-Spanish translation in particular is the main language combination of the PI’s expertise in institutional legal translation, which was a condition not only for designing the study but also for coordinating the selection of competent test validators and other aspects of implementation, together with the co-author of this study. Access to a large community of English-Spanish institutional translators would also be secured.

After several selection rounds, three candidate STs and their TTs were analysed in more detail in order to choose the one which would better meet all the above requirements and thus be most suitable (and ‘institutionally neutral’) for our testing purposes. The text finally selected is a report from a small common law country on legal implementation matters. This kind of descriptive text, including an outline of a national legal framework, is typically found across international institutions in the context of reporting obligations before specific bodies. Legal reports of several sub-types actually are the most common legal genre translated in such settings according to our preliminary textual mapping (Prieto Ramos and Guzmán 2021). More particularly, the text selected contains a description of civil and criminal procedures in cases related to intellectual property.

Several extracts of the text were selected to encompass the main features of the ST and the quality issues found in the TT, without compromising the coherence and understanding of the content. The title was shortened as ‘Issues on enforcement’, and it is followed by a section on ‘Civil judicial procedures and remedies’ and another one on ‘Criminal procedures’. No document symbol or other hint of the source was provided, and the ST and TT extracts were not modified. After several tests, and considering the error density for revision within the estimated timeframe of 75 minutes, the total length was set at 494 words.

In turn, the extracts selected were considered representative of features and difficulties of institutional legal translation from English, including references to basic concepts of law and legal procedure (e.g. ‘remedy’, ‘offence’, ‘evidence’, ‘hearing’, ‘witness summons’), legal actors (e.g. ‘magistrate’, ‘attorney-at-law’, ‘legal practitioner’, ‘Director of Public Prosecutions’), titles of legislation (e.g. ‘Civil Procedure Rules’, ‘Criminal Procedure Code’), court names (e.g. ‘High Court of Justice’, ‘Court of Appeal’), as well as legal phraseology (‘appear before the Court’, ‘reasonably and with probable cause’, ‘prejudicially affect’) and paraphrasing of several legal provisions. One of the segments that paraphrased legislation was particularly long (88 words) and convoluted. However, the extracts do not contain any genre or discourse convention requiring knowledge or research about any specific international institution for translation purposes.

The (human) translation of the text contains a high density of inadequacies, especially considering the quality expected of a professional translation. The most prominent issues are related to legal terminology, including the use of incorrect false friends (‘magistrado’ for ‘magistrate’ and ‘remedio’ for ‘remedy’) and other inappropriate translations of terms or phraseology such as ‘complaint’ in the context of criminal proceedings (translated as ‘reclamación’ instead of ‘denuncia’) or ‘appear before the Court’ (translated as ‘aparecer ante el tribunal’ instead of ‘comparecer’). The TT also contains several awkward reformulations that could be improved to enhance readability, as well as terminological inconsistencies, two punctuation mistakes and an inaccurate rendering of a segment that can be associated to general translation skills rather than LTC in particular. In total, 12 errors were considered of mandatory correction, including nine that signal the inability to grasp legal meaning or to identify or use established legal terminology, which, in turn, can be associated to insufficient LTC and thematic sub-competence in particular.

Overall, the TT can be considered of low quality and the result of inadequate LTC. It was most likely not revised or poorly revised by someone without the relevant LTC to spot and fix errors. The ST was probably perceived as a low-risk, low-priority text for TQA due to its limited scope with regard to a small country in the context of a regular monitoring procedure. Nonetheless, for the sake of reliable multilingual communication and legal certainty, the document at hand, as any other publicly available text with implications for a Member State and the conduct of institutional work, should not contain errors in any of the official languages. The text is therefore ideal to test to what extent translation professionals of various profiles are able to fill the TT quality gaps, above all, by spotting and correcting errors. This would be straightforward for any translator acquainted with basic concepts of law and legal procedures, without the need for specialisation in intellectual property law, procedural law or any other branch of law.

The revision task validation process (see Section 2.3) included several tests to confirm the nature of the TT errors and discriminate between mandatory corrections and other potential text quality improvements. Apart from the co-authors of this study, four other translators with experience in legal translation participated in the preparatory phase. An evaluation framework developed by LETRINT’s PI on the basis of a comprehensive analysis of professional, academic and institutional translation quality assessment approaches was applied to classify errors and their severity levels. This framework includes 14 error types within three categories (accuracy, linguistic correctness and terminological and functional adequacy^2^).

However, for the purposes of this study, the main focus is on errors associated to insufficient LTC, and on distinguishing between mandatory corrections and other revision actions (see Section 2.3 for more detail). To this end, as specified above, nine out of the 12 errors of mandatory correction found in the text were considered of a legal nature and grouped together as ‘legal content errors’ requiring adequate knowledge of legal subjects or legal discourses, regardless of the specific error type according to our taxonomy or any other quality metrics taxonomy. Five of the nine errors of a legal nature were of significant severity, while the rest were considered minor errors based on the preliminary tests. As a whole, the issues detected in the TT, including inappropriate terminology and phraseology, inaccuracies and inconsistencies, make it difficult to ascertain whether the translation is raw MT output or a poor human translation. In order to preserve the reputation of the institutional source of the TT, this source will not be revealed.

### 2.2. Professional profiles

Profile specifications were established according to our two central criteria for the comparison of revision performance among institutional translators: academic background and experience level. The first one was determined by the main field in which university studies were completed as a pathway to professional legal translation (degrees in law, translation or other areas), and whether such studies or any other additional training included legal translation or law courses. The various training pathways were grouped as follows:

- Legal or lawyer-linguist profiles (‘LL’): translation practitioners holding a law degree as their main qualification, including but not limited to those appointed as ‘lawyer-linguists’.^3^
- Legal translation practitioners formally trained as such (‘LT’): translators with an academic background in translation, including legal translation and law components.
- Qualified translators (‘T’): holders of a translation degree but with no specialisation in law or legal translation.
- Other translators (‘T0’): those with academic qualifications in other areas and no training in law or translation prior to entering professional practice as translators.

As regards experience, a distinction was made between two sub-groups per profile in order to measure the potential impact of longer experience levels on revision performance: those with three or more years of professional practice in legal translation (experience level 1) and those with less experience (level 2). For example, a ‘LL1’ translator belongs to the LL sub-group with experience level 1, while a ‘T2’ translator belongs to the ‘T’ group and has less than three years of professional experience in legal translation. Each level of translation experience was expected to include post-editing and some revision practice, not only of peers’ draft translations (or ‘other-revision’), but also of segments of previous human translations retrieved by translation memories.

The study primarily aimed at examining the differential impacts of training and experience on revision proficiency in legal translation. It had a particular focus on the comparison between LLs’ and LTs’ performance with that of other translators when reaching a minimum of three years of experience, which was considered a significant period for substantial ‘learning on the job’. This comparison would also contribute to empirically testing the rationale behind qualification requirements in ISO 20771:2020, which, as mentioned above, does not acknowledge the value of legal translation programmes as particularly relevant training pathways for professional legal translators. Professional profiles with a specialisation in law or legal translation were expected to show greater proficiency in detecting legal content errors (based on their presumed more-advanced acquaintance with legal subjects and texts), while more experienced translators were expected to perform better than participants with less experience within the same profile group.

The target number of participants for these profile clusters was set at between five and 10 for experience level 1, with control groups of less experienced translators of up to five participants. A call for participation in the study was disseminated across international organisations in late 2022 through the International Annual Meeting on Language Arrangements, Documentation and Publication (IAMLADP) network. A total of 29 in-house translators initially volunteered for the study. Based on the provisional figures for each profile group, a second round of participation requests was addressed to translation service managers in early 2023 with a view to recruiting additional candidates and balancing the size of the various cohorts. This was gradually achieved in the subsequent months until June 2023.

It became apparent that those with less specialisation or less experience in legal translation were less willing to participate, as they might feel more insecure about their proficiency. The recruitment process itself indirectly became an indicator of self-confidence and motivation levels for legal translation among translators of the different target profiles. Those who were specialised in legal translation (LL1s and LT1s) were the most eager to participate. In contrast, translators who were relatively new to the profession and had received no previous training in translation (LL2s and T02s) were the most reluctant to participate, despite the fact that we emphasised the interest to include them in the study and that compensation for participation was offered to all (see Section 2.3). The LL2 and T02 control groups thus had to be kept under five participants, as opposed to the other groups of between five and 10 translators. The priority for the research remained to gather valid data by making sure all participants paid the necessary attention to the revision test, so from a certain point, it would be somehow counter-productive to insist on adding more participants.

In spite of the recruiting challenge, typical for this kind of study, the total of 47 participants, which subsequently became 44,^4^ was considered highly satisfactory considering the total population of English-Spanish institutional translators and their time constraints. The unique sample was extremely valuable for the comparison of more specialised legal translators (LL1s and LT1s at 10 participants each) with less specialised profiles (a combined total of 10, sub-divided into five T and five T0 translators) (see Table 1).

**Table 1.** Number of participants per profile group.

	1	2	Total
LL	10	2	12
LT	10	5	15
T	5	5	10
T0	5	2	7
Total	30	14	44

The distribution between institutional affiliations was also deemed very satisfactory and representative of the diversity of language services and staff profiles in international institutional settings, with 17 from four IGOs (including seven from the United Nations), 13 translators from four major EU institutions and 14 freelancers or temporary staff regularly working for international organisations. To our knowledge, the sample of participants is the largest and most diversified to date for this kind of empirical research in institutional translation studies, even though the size of two control groups of less experienced translators finally stood at two rather than five participants each. While these smaller LL2 and T02 groups would be less statistically significant, they would still provide valuable data to compare patterns with the more experienced groups.^5^

### 2.3. Revision task and data processing

Apart from the ST and TT selected, the instructions and the evaluation framework to be provided to participants, a questionnaire was developed to gather information on their academic and professional background, and on their overall impression of the translation quality. This last part was to be completed after the fine-grained revision exercise in order to cross the revision scores with a more holistic translation evaluation by each participant, including an assessment of the entire TT, its potential machine or human origin, and the three aspects evaluated through the error analysis: accuracy, linguistic correctness and terminological and functional adequacy. This questionnaire also included a question on the time devoted to the revision. Finally, a guide was also prepared to facilitate the use of the platform selected for the revision task.

**Figure 1.**
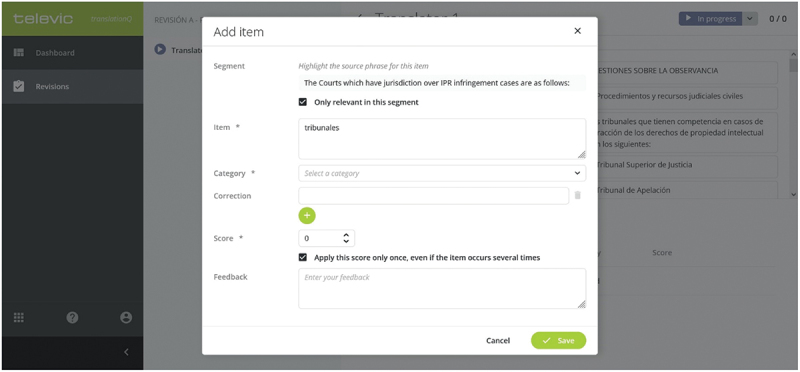
Screenshot of TranslationQ’s interface.

Televic’s TranslationQ was identified as the most suitable tool to implement the test, as it offers the possibility of uploading a customised error typology, and it ensures a simplified revision process through which users click on the relevant segments (in a bitext display) and select both the error type and the severity level from pre-configured lists (see screenshot in Figure 1). This guaranteed harmonised conditions for all participants and a reduced risk of facing technical problems, thus increasing the attention dedicated to revision itself.

Four qualified legal translators participated in the test validation phase in the autumn of 2022. They not only completed the revision exercise as mentioned in the previous section, but were also asked to assess the protocol, in particular, the platform’s ease of use and the clarity of the instructions and the accompanying documents. Their feedback was used to refine a few aspects of this material.

Participants in the revision test were initially asked to complete an online survey in which they indicated (1) their contact information and availability, (2) whether they were willing and allowed to accept compensation for their participation (in the form of a symbolic remuneration or a voucher), especially if time had not been allocated by their supervisors for the task, and (3) their consent to the anonymised use of their input for research purposes. The revision brief and other instructions for the revision task were then sent by email, together with the customised TranslationQ user guide and the evaluation framework.

The brief identified the text as an extract from a national report submitted to institutional bodies and translated for the entire Spanish-speaking world. It underlined that the revision was expected to correct errors rather than introducing stylistic or other optional improvements. It also specified that (1) there was no need to adhere to any institution-specific terminology, but conform to the appropriate legal terminology and phraseology in connection with the subject-matter; and (2) all information sources deemed of relevance could be consulted, as long as the revision work was done individually.

No time limit was set for the revision, but a maximum of approximately 75 minutes was recommended for the task and 10–15 minutes for the final online questionnaire (link provided in the same email). Participants were asked to keep track of the total time devoted to the revision, excluding any breaks. The total revision time would then be inserted in the final questionnaire, together with an identification code assigned to each participant to anonymise their inputs.

The revisions were conducted between late 2022 and mid-2023. The following information was recorded for each revision: a unique alphanumeric identifier, the revisor’s identification code and profile, the affected segment (i.e. each sentence requiring revision), the corrected content, the error type and severity, its proposed correction and comments on the correction, if any. For the sake of uniformity and comparability, when a participant marked the same correction more than once (i.e. because the error was repeated in the TT), only the first iteration was counted.

A total of 1424 iterations were initially registered. However, after a double validation process, the input from three participants had to be discarded from the analysis due to an obvious lack of revision attention which produced potential data distortions for the corresponding profile groups. This risk derived from the exceptionally short time devoted to the exercise (less than half the recommended time) and the limited, even negligible, number of corrections that resulted (less than one third of the average per participant).

The remaining 1394 iterations were thoroughly processed. In line with the research aims and the revision brief, the analysis of revision interventions focuses on mandatory *versus* unnecessary corrections, i.e. indicators of the ability to ensure that the final TT is correct and error-free. More specifically, average correction scores were calculated for each profile group and error group (legal content and other errors), distinguishing between the following indicators:

- Total number of corrections or revision interventions, actions or iterations.
- Justified revisions, i.e. ST issues correctly spotted, including mandatory corrections identified as such in the validation phase (nine out of these 12 are of a legal nature) and other revisions that clearly improve the TT (e.g. changes to awkward formulations that are grammatically acceptable but hinder readability or idiomaticity).
- Unnecessary revisions, including instances of ‘hyper-revision’ or modifications with a neutral impact on the TT quality (in line with metalanguage in the field; see e.g. Horguelin and Brunette 1998; Künzli 2006), and instances of ‘over-revision’ with a negative impact on the TT (i.e. new errors introduced).
- Missing mandatory corrections, i.e. overlooked errors or instances of ‘under-revision’.

The average severity levels assigned per profile group and error group were also calculated, but they fall outside the scope of this study and will not be examined here. Likewise, this study will not investigate how participants classified the different errors according to the taxonomy applied; nor will it provide detailed measurements of the quality or potential added value of the specific changes they introduced, other than the above distinction between justified, unnecessary (or at times inappropriate) and missing corrections.

## 3. Results

Our analysis focuses on indicators of the ability to detect and fix errors with the aim of producing an error-free TT as a revision priority: (1) justified corrections that improve the translation, including mandatory corrections, as a positive competence score; and (2) missing mandatory corrections (undetected errors) and over-corrections (errors introduced) as negative competence scores. The latter were identified as a sub-group of unnecessary corrections, which otherwise include hyper-revisions of a neutral impact on the TT. The combination of the two negative competence scores results in the total number of errors remaining after the revision process, which, in turn, is a key sign of revision efficiency gaps. These scores will be compared per profile group and error group, and will then be cross-checked with revision times and the results of a more holistic translation evaluation by the participants.

### 3.1. Justified corrections and missing mandatory corrections

**Figure 2.**
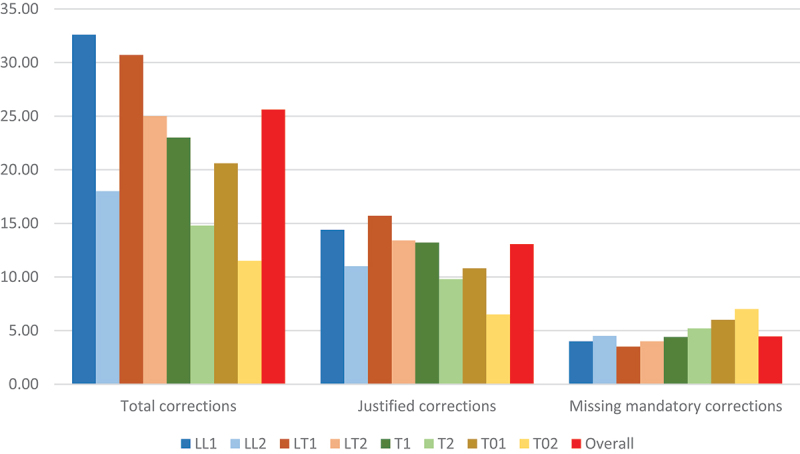
Average number of total, justified and missing corrections per profile group.

In line with our hypothesis, translators with more specialised profiles performed better in detecting and correcting errors, especially of a legal nature. LT1s stand out with the highest **justified revision scores** for both legal content corrections and other corrections, and a total average of 15.70 justified corrections, including 8.50 of the 12 errors of mandatory correction, and 3.50 undetected errors (see Table 2 and Figure 2). They are followed by LL1s and LT2, with extremely similar results in terms of mandatory corrections (8 spotted *versus* 4 overlooked errors) and only a difference of one iteration of additional justified revision by LL1s. The scores of the three top-performing cohorts more than double those of the weakest group in terms of legal translation training and experience, T02s, with an average of 6.50 justified revisions and 7 missing corrections.

**Table 2.** Average correction scores per profile group.

	All	Justified (of which mandatory)	Unnecessary (% over total)^6^	Missing mandatory
LL1	32.60	14.40 (8.00)	18.20 (55.83%)	4.00
LL2	18.00	11.00 (7.50)	7.00 (38.89%)	4.50
LT1	30.70	15.70 (8.50)	15.00 (48.86%)	3.50
LT2	25.00	13.40 (8.00)	11.60 (46.40%)	4.00
T1	23.00	13.20 (7.60)	9.80 (42.61%)	4.40
T2	14.80	9.80 (6.80)	5.00 (33.78%)	5.20
T01	20.60	10.80 (6.00)	9.80 (47.57%)	6.00
T02	11.50	6.50 (5.00)	5.00 (43.48%)	7.00
Overall	**25.61**	**13.07 (7.55)**	**12.55 (48.98%)**	**4.45**

T1s (13.20 average score of justified corrections and 4.40 missing corrections) and LL2s (averages of 11 and 4.50, respectively) registered values that are close to the overall averages for all the participants. T2s and T01s ranked next with a minor positive difference of mandatory corrections for the first group (6.80 *versus* 6), while the second group had, on average, an additional justified revision (total of 10.80 *versus* 9.80). Finally, the two participants with no formal training in translation or law and limited experience in legal translation (T02), performed worse than the other groups, with an average of 7 (out of 12) undetected mandatory correction errors.

**Figure 3.**
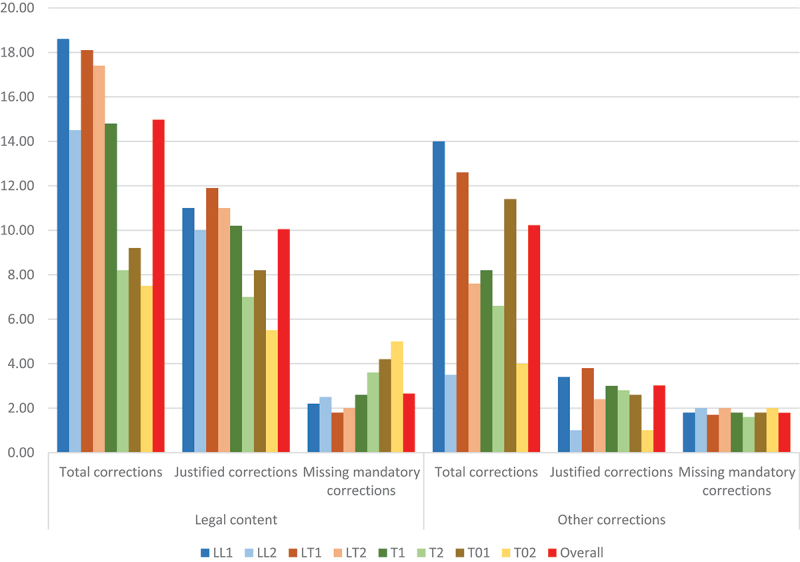
Average number of total, justified and missing mandatory corrections per profile group and error group.

More experienced cohorts systematically show better performance than less experienced translators, with an average of 37.23% more justified corrections and 0.7 more mandatory corrections. However, the variations are less marked than between profile groups based on academic background. At equal minimum **experience levels**, those formally trained in legal translation outperform those with academic backgrounds in law, translation (but no specialisation in legal translation) and other fields. The positive impact of legal translation experience within each profile group ranges between 2.30 additional justified corrections on average for LT1s as compared to the LT2 control group, and 4.30 iterations of positive difference for T01s *versus* T02s, with T1-T2 and LL1-LL2 divergence standing at 3.40. These competence gains are more pronounced in legal content corrections or more general corrections (see Table 3 and Figure 3) depending on the legal or translation specialisation gaps in the academic background of each group (i.e. more marked improvement in linguistic aspects in the case of LLs, legal aspects among Ts, and both aspects in the case of T0s with no specialisation in translation or law). This clearly suggests that experience can serve, at least partially, to fill certain training deficits.

**Table 3.** Average correction scores per profile group and error group.

	Legal content corrections				Other corrections			
	All	Justified (of which mandatory)	Unnecessary	Missing mandatory	All	Justified (of which mandatory)	Unnecessary	Missing mandatory
LL1	18.60	11.00 (6.80)	7.60 (40.86%)	2.20	14.00	3.40 (1.20)	10.60 (75.71%)	1.80
LL2	14.50	10.00 (6.50)	4.50 (31.03%)	2.50	3.50	1.00 (1.00)	2.50 (71.43%)	2.00
LT1	18.10	11.90 (7.20)	6.20 (34.25%)	1.80	12.60	3.80 (1.30)	8.80 (69.84%)	1.70
LT2	17.40	11.00 (7.00)	6.40 (36.78%)	2.00	7.60	2.40 (1.00)	5.20 (68.42%)	2.00
T1	14.80	10.20 (6.40)	4.60 (31.08%)	2.60	8.20	3.00 (1.20)	5.20 (63.41%)	1.80
T2	8.20	7.00 (5.40)	1.20 (14.63%)	3.60	6.60	2.80 (1.40)	3.80 (57.58%)	1.60
T01	9.20	8.20 (4.80)	1.00 (10.87%)	4.20	11.40	2.60 (1.20)	8.80 (77.19%)	1.80
T02	7.50	5.50 (4.00)	2.00 (26.67%)	5.00	4.00	1.00 (1.00)	3.00 (75.00%)	2.00
Overall	14.98	**10.05 (7.00)**	**4.93 (32.93%)**	2.00	10.23	**2.50 (1.16)**	**7.20 (70.44%)**	**1.84**

In fact, a closer examination of the errors corrected reveals that the main inter-profile score variations are found in the **corrections of legal content errors**. Justified corrections of this nature decrease among those who have been less exposed to legal texts and legal translation in their training and translation careers (down from averages of 11.90 for LT1s and 11 for LL1s and LT2s, to 7 for T2s and 5.50 for T02s), while the missing legal content corrections increase (from 1.80 for LT1s and 2 for LT2s to 4.20 for T01s and 5 for T02s). The fact that T0s did not detect approximately half of the legal content errors in the TT can only derive from insufficient legal knowledge and inadequate LTC, especially when considering that the undetected errors typically included some of the most serious ones, such as the misleading renderings ‘magistrado’ and ‘reclamación’ mentioned earlier. These were overlooked by all T0 translators except for one T01, as compared to much lower missing correction rates among the other groups.

Overall, this led to a significant difference in the quality of the final revision. The same gaps were found, albeit to a lesser extent, among T2 translators. Again, T1 translators seem to have compensated for training gaps in law or legal translation with professional experience in the field, to the point that their legal content revision scores are almost identical to those of law graduates with limited experience in translation (LL2s). At the same time, T1s performed better when dealing with other translation issues, which accounted for the comparative difference in this case.

As for **corrections of a more general nature** among the other groups, they range between the lowest average of one correction for the least trained and experienced in translation (LL2s and T02s) and 3.80 justified corrections for LT1s. However, the actual inter-group difference between missing correction scores is minimal, with averages ranging between 1.60 and 2 undetected non-legal errors. This is explained by the general difficulty in spotting the two minor punctuation mistakes in the translation. With one exception, all participants overlooked or simply tolerated at least one of them.

### 3.2. Unnecessary corrections

As regards the other neutral or more negative revision indicators, the average rate of unnecessary corrections is 4.93 for legal content errors (or 32.93% of total corrections in this category) and 7.20 (70.44%) for other issues. This is explained by the high proportion of revision iterations that could not be considered error corrections but stylistic changes with no significant positive impact in context, for instance, using the more technical verb ‘emplazar’ instead of ‘requerir’ in ‘documento en el que se requiere a un testigo que comparezca ante el tribunal’ (‘document requiring a witness to attend court’), or using ‘estará’ instead of ‘está’ in ‘esa parte está obligada a […]’ (‘that party is obliged to […]’). This contrasts with justified (positive impact) revisions such as replacing the verb ‘poner de manifiesto’ with ‘facilitar’ or ‘comunicar’ in ‘poner de manifiesto todos los documentos’ (‘disclose all documents’), or using ‘alguien’ instead ‘cualquier persona’ in ‘creer que cualquier persona ha cometido un delito’ (‘believe that an offence has been committed by any person’), even if the initial TT segment may be considered somewhat unidiomatic but not necessarily an error.

LL1s introduced the highest number of unnecessary corrections, with averages of 7.60 for legal content corrections and 10.60 for other corrections. Otherwise, the patterns for the other groups align with those of justified corrections of legal content errors, with those trained in legal translation ranking second (6.40 for LT2s and 6.20 for LT1s), and T1s (4.60) and LL2s coming next (4.50). Finally, the least specialised profiles (T2s and T0s) introduced few unnecessary legal content changes, between one and two per group on average. In other words, the tendency to question and modify TT segments increases according to the level of familiarity (and confidence) with both legal subjects and legal translation. The shares of unnecessary changes over the total number of revisions also reflect this pattern, with the highest proportion of almost 41% among LL1s, around 35% for LTs and 31% for T1s and LL2s.

Overall, less experienced groups introduce fewer unnecessary changes than the more experienced groups in each profile, which points to less critical questioning of the TT or more hesitant attitudes towards proposing potential improvements. In the case of unnecessary corrections of a non-legal nature, variations seem to be particularly influenced by experience in legal translation, with systematically higher scores for the more experienced, including averages of 8.80 for LT1s and T01s, and 5.20 for T1s, and particularly low scores for the least linguistic profiles (2.50 for LL2s and 3 for T0s, in line with their low scores of justified corrections in the same non-legal category).

These figures include a marginal number of over-corrections, i.e. unnecessary corrections which actually introduced errors. As expected of professional translators, these errors are rare, only 18 out of 1384 iterations, and generally account for marginal proportions of less than 5% of total corrections per profile (see Table 4). A total of 30 out of the 44 participants introduced no errors, and no single translator made more than two over-corrections. The less experienced groups introduced almost no errors, except for a minor linguistic over-correction by a T2 participant. This certainly derives from a more cautious approach to making changes in comparison with more experienced (and self-confident) profiles, as mentioned above. The highest score of over-corrections is that of T01s (4.85%) in particular due to four minor reformulation mistakes of a general nature (punctuation and inadequate lexical alternatives), which actually represent 7.02% of total revisions for this category and profile group.

**Table 4.** Over-corrections and errors remaining in revised TT per profile group.

	Total over-corr.		Legal content over-corr.		Other over-corrections		Errors remaining in revised TT
	Corr.	Avg. (% of total corr.)	Corr.	Avg. (% of total corr.)	Corr.	Avg. (% of total corr.)	
LL1	7	0.70 (2.15%)	6	0.60 (3.22%)	1	0.10 (0.71%)	4.70
LL2	-	-	-	-	-	-	4.50
LT1	2	0.20 (0.65%)	1	0.10 (0.55%)	1	0.10 (0.79%)	3.70
LT2	-	-	-	-	-	-	4.00
T1	3	0.60 (2.61%)	3	0.60 (4.05%)	-	-	5.00
T2	1	0.20 (1.35%)	-	-	1	0.20 (3.03%)	5.40
T01	5	1.00 (4.85%)	1	0.20 (2.17%)	4	0.80 (7.02%)	7.00
T02	-	-	-	-	-	-	7.00

The case of legal content over-corrections by LL1s (6 out of 7 over-corrections) is also worth some attention. While this group is expected to conduct thorough revisions of legal aspects, some errors may result from the abovementioned propensity to ‘take more risks’ and propose improvements often based on knowledge of the home legal system and legal language. This can sometimes lead to inadequate adaptations, such as replacing ‘Reglamento de Procedimiento Civil’ with ‘Normas de Enjuiciamiento’ for ‘Civil Procedure Rules’, or overshadowing some semantic nuance of the source legal system, as illustrated by a reviser who mistakenly introduced ‘representante legal’ instead of ‘abogado’ for ‘attorney-at-law’. In turn, this type of error usually denotes insufficient proficiency of legal translation methodological or strategic sub-competence (see Prieto Ramos 2024), and, in particular, difficulty in applying comparative legal analysis and appropriate translation procedures to ensure TT communicative adequacy. This is precisely a key component of legal translation methodology developed through legal translator training.

As pointed out earlier, the combination of missing mandatory corrections and over-corrections provides a relevant indicator of revision efficiency gaps, as the priority of the task is to produce an error-free text. When factoring this in (see Table 4), the performance of LTs stands out more clearly than when only justified corrections are considered (Table 1), and, in effect, resolves the tie between LL1s and LT2s, in favour of the latter. LL1s are more penalised by over-corrections than other top-performing groups, even if this data must be read in conjunction with the positive indicators. The ranking of negative scores is otherwise globally consistent with the competence indicators previously examined.

### 3.3. Revision time and overall efficiency

The average times devoted to the revision task reveal that legal profiles (average of 100 minutes for LL2s and 99.50 for LL1s) spent more time on the task than the other groups (Table 5). However, considering the revision scores, the less experienced LL2 translators used the time less fruitfully (probably due to the need to make more verifications and more cognitive effort), while LL1s seem to have devoted more time to unnecessary corrections than any other group.

**Table 5.** Average time devoted to the revision task per profile group (in minutes).

	1	2	Overall
LL	99.50	100.00	99.75
LT	82.40	75.00	78.70
T	72.00	72.00	72.00
T0	83.00	49.00	66.00
Overall			82.43

The most efficient groups in terms of time management are LT1 and LT2s, with a task duration close to the overall average and the most satisfactory correction indicators. The better performance of LT1s entailed the use of a few more minutes than LT2s (average of 82.40 *versus* 75), but their results share a common pattern. The greater time efficiency shown by LTs in general might be explained by their expected familiarity with comparative legal analysis, legal translation techniques and specialised search tools through training, which normally contributes to time gains. The practiced ability to conform to translation and revision specifications (e.g. considering the purpose and focusing on error correction in this case) may also be a factor.

Apart from the longer time registered by LLs, a correlation can be generally established between the time average and the revision results, with two additional exceptions. T2s spent as much time on the revision as T1s but with worse results, while T02s used, on average, 11 more minutes than T1s and as much time as LT1s, but delivered more disappointing results. All in all, our findings corroborate that the less experienced revisers need more time for revision actions, a sign of more limited proficiency, and those not trained in translation (LLs and T0s) are comparatively less efficient.

As noted by Künzli (2007), adding more time for the revision does not necessarily lead to better final quality. Our study shows that the key to revision efficiency lies, above all, in the reviser’s competence. In fact, as illustrated by the indicators for the strongest and weakest performances (Table 6), the three best revisions (in terms of justified corrections and errors remaining in the final TT) were conducted in less time than the worst one. The average time for the five top revisions (77.8 minutes) is only 8 minutes longer than the average for the five revisions at the bottom of the ranking (69.6). The best revisions were also the most time-efficient.

**Table 6.** Revision scores and times for the best and worst revisions.

Ranking	Participant	Justified corr. (incl. mandatory)	Unneces. corr.	Missing corr.	Over-corr.	Remaining errors in TT	Time
1	LT1–3	18 (11)	22	1	-	1	70
2	LT2–2	21 (10)	11	2	-	2	70
3	LT1–10	19 (10)	22	2	-	2	84
4	LL1–3	18 (10)	22	2	-	2	90
5	LT1–7	17 (10)	7	2	-	2	75
40	T2–3	9 (6)	8	6	1	7	75
41	T01–3	17 (5)	14	7	1	8	90
42	T02–1	7 (4)	3	8	-	8	40
43	T02–2	6 (6)	7	6	2	8	58
44	T01–4	6 (4)	1	8	-	8	85

### 3.4. Holistic quality assessment

**Figure 4.**
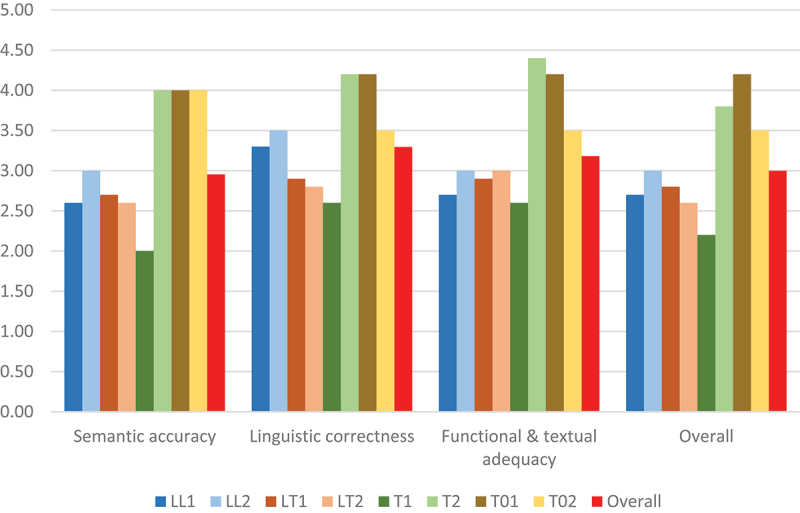
TT holistic assessment per profile group (average values).

The global assessment of the TT conducted after the revision task yielded very relevant information about the participants’ perceptions of the revised translation and how these compare with their revision patterns. Indeed, the results of the more holistic analysis (see Table 7 and Figure 4) generally match those of the fine-grained error-based approach. With the exception of the T1 group, the more specialised profiles (LTs and LLs) were more critical about the quality of the TT than the less specialised profiles (Ts and T0s). Whereas T01 and T02 participants’ assessment of accuracy, linguistic correctness and terminological and functional adequacy reaches values between 3.50 and 4.20 points in a scale of 0 to 5 (i.e. mostly acceptable quality), LL and LT participants’ assessment of the TT is globally negative (between bad and borderline averages) and only exceeds 3 points in the case of LLs’ assessment of language and style. This is the main difference with the more ‘linguistic’ profiles of LTs, also in line with their respective correction scores.

**Table 7.** TT holistic assessment per profile group (average values).

	LL		LT		T		T0		Overall
	1	2	1	2	1	2	1	2	
Semantic accuracy*	2.60	3.00	2.70	2.60	2.00	4.00	4.00	4.00	**2.95**
Language and style	3.30	3.50	2.90	2.80	2.60	4.20	4.20	3.50	**3.30**
Terminological and functional adequacy	2.70	3.00	2.90	3.00	2.60	4.40	4.20	3.50	**3.18**
Overall	2.70	3.00	2.80	2.60	2.20	3.80	4.20	3.50	**3.00**

*Scale: 0-1 = unacceptable/very bad; 2 = poor/bad; 3 = borderline; 4 = acceptable/good; 5 = excellent/very good.

The results for the less experienced control groups are globally similar to those of their more experienced peers with a comparable academic background, but not necessarily less critical (e.g. overall assessment of 2.60 for LT2s *versus* 2.70 for LT1s). The major intra-profile disparity is found among Ts because of T1s’ particularly negative views (between 2 and 2.60 points for the various aspects), as opposed to T2s’ positive impressions (3.80–4.40 points). T1s’ assessment is actually the most negative of all the groups with regard to all aspects evaluated, and represents the only mismatch with the correction scores in comparison with other profiles. While T1s obtained mid-range revision results and can be considered the fourth best-performing profile group after LT1s, LT2s and LL1s, their negative TT perceptions were not as consistently reflected in effective correction actions. This points to an ability to perceive low quality but more difficulties or more limited specialised competence to improve legal translation quality in particular. At the other extreme, the weakest performers within T2 and T0 groups do not even perceive low quality.

The comments provided in the holistic assessment questionnaire are illustrative of these disparities (see examples in Table 8). Since they are all made by translators working for international organisations, these qualitative insights remind us of the potential consequences of assigning legal translation or revision work to professional translators with a low specialisation in this area. The comments are also indicative of the genuine attention devoted by the participants to the revision exercise.

**Table 8.** Illustrative comments about the quality of the revised TT (our translations).

Predominantly negative	Predominantly positive
LL1–1: I detected several issues of semantic accuracy and internal consistency (the same term is translated in multiple ways, which is unacceptable for a short text), and low thematic competence (deficiencies of background legal knowledge).	T01–1: Although I indicated several potential improvements, there are barely any substantial errors that could affect the correct comprehension of the text.
LL1–7: In general, the target text reflects lack of knowledge of basic legal notions, as it has been drafted closely following English phraseology. This lack of knowledge could have been overcome through a minimum amount of terminological and conceptual research, but this was apparently not done. As a result, the target text is comprehensible but of a rather poor quality.	T01–2: Despite some inconsistencies, there are no serious mistranslations and the target text is easily readable.
LT1–3: In general, the target text lacks in textual and functional adequacy. It does not conform to the phraseology and conventions of its genre. There are also several terminological and phraseological inconsistencies. The final text does not seem to have been prepared on the basis of the necessary prior research.	T01–4: The issues corrected do not hinder text comprehension. The translation seems to be the work of a specialised translator.
LT1–8: In my opinion, terminological accuracy and register adequacy are mediocre, and there are some ortho-typographic errors related to the use of caps and punctuation. I have also identified mistranslations which, given the type of text, are particularly serious due to their potential legal implications.	T01–5: The target text is perfectly understandable and fluid to a certain extent, even if translation solutions are not totally systematic and one sentence reads rather intricate.
LT2–4: The quality of the target text is very poor. There are terminological errors, seriously misleading false friends, multiple terminological and phraseological inconsistencies, calques from English and fluidity issues that considerably hinder target text comprehension.	T2–3: I find the target text easy to read in general. Given its descriptive nature and low level of specifics or specialisation, the terminology is appropriate for the intended register. There are some minor consistency issues, but they do not hinder text comprehension in context.

Finally, when asked if they had the impression that the TT could have been produced by an MT system (Table 9), approximately two thirds of participants replied positively. The results did not yield any clear pattern per profile group, which further corroborates that the issues found in the TT made it very difficult to assert whether it was MT raw output or a poor human translation.

**Table 9.** Participants who considered that the TT was produced by an MT system (proportions per profile group).

	1	2	Overall
LL	50.00%	50.00%	50.00%
LT	90.00%	100.00%	93.33%
T	60.00%	60.00%	60.00%
T0	20.00%	100.00%	42.86%
Overall			**65.91%**

## 4. Discussion and conclusions

Our correlational analysis of positive and negative correction scores and profile specialisation according to academic background and legal translation experience suggests that the differential impact of legal translator training on LTQA performance is more marked than that of professional experience in the field. Translators holding university degrees in translation with a specialisation in legal translation stood out as the best-performing, as compared to groups with other academic backgrounds but similar experience levels. Law graduates with a good command of languages ranked second at each experience level, followed by trained translators with no specialisation in legal translation and, finally, translators with other backgrounds. As an indicator of early career expertise following academic training, the scores of justified corrections for the best group at the lower level of experience (LT2) were more than double those of the weakest (T02).

Legal translation experience has a positive impact on performance in every profile group (37.23% more justified corrections on average among the more experienced cohorts), but it is more limited than that of training specialisation. However, the gradual benefits of experience are more marked precisely within groups lacking legal or translation training specialisation, as experience seems to partially compensate for such training gaps. The competence gains are accordingly more tangible in terms of linguistic revisions among LLs, legal content corrections in the case of Ts and both aspects in revisions by T0s (i.e. those with no specialisation in translation or law). As a result, law graduates (LL2s) and trained translators without legal specialisation (T2s) converge towards the scores of the top-performing LT groups, while T01s still lag behind despite the benefits of professional practice. In other words, experience proves to be more of an ‘equalising factor’ in competence development when the foundations in translation or law are solid.

As regards the variations of legal content corrections and other corrections between profile groups, another key aspect examined in the study, the first type of corrections accounts for the most significant difference in performance. Translators who have been more exposed to legal texts and legal translation in their training and translation careers detect and fix legal content errors more systematically, and those who are less familiar with legal topics overlook them more often, including some of the most serious mistakes. In contrast, the scores for other corrections of a more general nature do not vary so significantly between profile groups, even though the least trained and experienced in translation (LL2s and T02s) correct linguistic issues less efficiently. In sum, these correction patterns point to the crucial role played by legal domain and legal translation specialisation in correcting errors of a legal nature or requiring adequate knowledge of legal subjects or legal language, which are distinctive components of legal translation competence and LTQA.

In order to complete the comparison of revision performances and efficiency per profile group, the above scores were examined in conjunction with revision times and patterns of unnecessary corrections, including hyper-revisions of a neutral impact on the final TT quality and over-corrections with a negative impact. More experienced translators appear more confident to introduce unnecessary changes, especially more experienced LLs (and this entails the risk of actually introducing some errors), while less experienced translators overlooked more errors but were less inclined to propose unnecessary changes and made almost no over-corrections. While some time-consuming unnecessary revisions may have been intended to improve style or readability, the priority of any revision (and of the proposed revision brief in this case) is to produce an error-free TT. Indeed, over-corrections were marginal. However, it is worth noting that LL1s introduced some legal semantic inaccuracies that can be linked to the inadequate application of legal translation techniques (i.e. core strategic or methodological sub-competence).

When factoring in the number of errors remaining in the revised TT (i.e. the addition of missing mandatory corrections and over-corrections as indicators of revision inefficacy), as well as the time spent on the revision task, and not only the justified correction scores, the performances by LT1s and LT2s stand out as the most efficient. This is followed by LL1s (who spent more time on unnecessary changes), T1s and LL2s. Finally, T2s and T0s delivered the most unsatisfactory revisions. The small T02 control group spent the shortest time on the task, and showed the most limited ability to detect errors and improve the TT. Unlike any other group, the number of errors overlooked by these translators was actually higher than their justified corrections.

The results of the subsequent holistic translation quality assessment by the same participants were globally consistent with their correction patterns, except for T1s’ particularly critical views of the TT. In addition, the exercise illustrated the extent to which a human translation of low quality can often be mistaken for raw MT output, thus blurring the distinction between revision and post-editing in practice. In turn, this reinforces the intended value of the exercise as a way of testing the core translation strategic and thematic sub-competences underpinning LTQA more broadly.

While our findings on revision performance cannot be generalised as precise markers of legal translation competence levels, they can be considered fairly indicative of degrees of expertise in this field. Revision is not only a key TQA activity, but also an increasingly relevant part of translation production itself and of translators’ skillset in light of the diversified human and machine inputs embedded in hybrid reuses of previous translations. When translating a ST or ensuring the quality of a previously translated text, the translator relies on a common core of methodological and domain-specific competence components to achieve TT adequacy.

Given the methodological design of the study and the unique size and diversity of its sample, and despite the more limited statistical significance of two of the less experienced control groups, our results provide new evidence of the added value of legal translator training for LTQA. The findings attest to the advantages of acquiring methodological competence specifically adapted to legal translation as the basis for efficiently applying thematic competence (the other crucial and distinctive competence component tested through our exercise) and other linguistic and instrumental skills.

In fact, the results empirically support previous considerations on the potential strengths and weaknesses of the various academic backgrounds examined (Prieto Ramos 2011, 19), in particular, how law graduates are expected to draw on more advanced legal knowledge (with a focus on national law but not necessarily so on other legal systems or comparative law), while translation graduates are trained for developing all translation competence components to be applied to specific domains. Merging the most relevant aspects of these backgrounds into a solid legal translation specialisation, rather than necessarily combining a translation and a law degree, emerges as an effective pathway to building professional expertise in legal translation. In sum, it should be no surprise that legal translators specifically trained to work in this field make good candidates for legal translation, as illustrated by our study.

By the same token, our findings suggest that the qualification specifications in ISO 20771:2020 are considerably arbitrary and do not seem to be supported by evidence. Translation degrees, and legal translator training programmes in particular, are inexplicably devalued in this standard: (1) by not acknowledging the specificity and relevance of legal translator training for legal translation practice (would you similarly ignore the benefits of a specialisation in cardiology when looking for a cardiologist rather than a general practitioner?); (2) by considering translation degrees as less relevant than law degrees for legal translation (as reflected in the additional requirement of a postgraduate degree in law, as opposed to no training in translation in the case of law graduates); and (3) by degrading the value of translation degrees, regardless of specialisation, to that of degrees in any other field (except for legal studies).

Consequently, some of the most competent LT participants in our study (with a specialisation in legal translation but no degree in law) would not be able to meet the ISO 20771:2020 requirements before reaching five years of professional experience, while LL2s with mid-range scores would be compliant when reaching three years of legal translation experience. In practice, top-performing LTs without a postgraduate diploma in law and less than five years of experience are considered by the ISO standard as insufficiently qualified for professional legal translation, to the same extent as the weakest performers in our study, i.e. those without law or translation qualifications (T0s). Given the emphasis on such requirements for individual legal translators’ compliance, this standard appears as a missed opportunity to have a more refined LTQA tool, including institutional settings. It contains inaccurate information about institutional legal translation, and it is inconsistent with the standing of translation degrees within the broader ISO 17100:2015 for translation services (ISO 2015) and with established practice and requirements for legal translation at international organisations.

The evidence outlined here can also prove highly relevant for profile classifications in the context of recruitment processes, risk analyses and workflow management, for example, when establishing the requirements for specific competitions or when assigning legal translation or revision jobs to the most suitable combination of translator or reviser profiles in each scenario (see also Allman 2006; Parra Galiano 2021). As elicited by our research, insufficient specialisation can lead to a poor translation being regarded as acceptable if the reviser is not more competent than the original translator (or MT system), thus potentially damaging the reputation of a language service or an organisation, on top of the possible legal implications. The increasing integration of several forms of translation reuse in today’s workflows has accentuated the added value of specialisation and the need for translation excellence in institutional settings in particular (see Prieto Ramos 2024). Translator competence is the first pillar of any sound TQA policy and its efficient implementation, and, as proven by this study, specialised training is a determining factor in building the required translation expertise.

## Notes

1. https://transius.unige.ch/letrint.

2. The first category concentrates on content and semantic nuance (e.g. unjustified omissions or additions and incorrect data), the second is about language mechanics and style (e.g. grammar, punctuation, spelling, register and fluency issues), and the last one focuses on conformity to domain or institutional conventions, consistency and decision-making adequacy from a functional perspective (e.g. terminological error or inconsistency, non-conformity to genre convention and inappropriate rendering considering the TT purpose). As for severity levels, a distinction is made between minor, significant, major and critical errors depending on their impact on content, comprehension, fluency, text function and usability. A short version of the ‘Framework for translation quality metrics’ can be accessed at: https://transius.unige.ch/en/research/letrint/quality-metrics. The rationale behind it falls beyond the scope of this study.

3. Contrary to what is inaccurately indicated in Annex B of ISO 20771:2020 (ISO 2020, 18–19), a law degree is not required as a general rule to translate legislation in the EU institutions or to translate legal texts in IGOs, but it is one of the qualifying degrees for recruitment (see Prieto Ramos and Guzmán 2022). The CJEU is the only EU institution where ‘lawyer-linguists’ (with a law degree) systematically work as legal translators, but they typically translate court documents, i.e. case-law and related texts, rather than legislation. In the other EU institutions, lawyer-linguists contribute to quality assurance and legal advising during the legislative process, but they do not generally translate. They are not ‘expected to have experience in translating and revising legislation’ to be recruited as asserted by the ISO standard (ISO 2020, 19).

4. These included some translators who were temporary staff without a permanent affiliation to a single organisation, but whose experience, often at several institutions, was extremely relevant.

5. Overall, on average, the participants of experience level 1 estimated that they had 12.6 years of experience in legal translation and 7.6 in ‘other-revision’ in this area, as opposed to 0.8 and 0.5 years, respectively, in the case of participants of experience level 2.

6. Including over-corrections or errors introduced (see Table 3).
